# *CsCER6* and *CsCER7* Influence Fruit Glossiness by Regulating Fruit Cuticular Wax Accumulation in Cucumber

**DOI:** 10.3390/ijms24021135

**Published:** 2023-01-06

**Authors:** Xiaofeng Liu, Xinshuang Ge, Jingbo An, Xingwang Liu, Huazhong Ren

**Affiliations:** 1 College of Horticulture, Qingdao Agricultural University, Qingdao 266109, China; 2 Engineering Research Center of Breeding and Propagation of Horticultural Crops, Ministry on Education, College of Horticulture, China Agricultural University, Beijing 100193, China

**Keywords:** cucumber, fruit glossiness, fruit cuticular wax, *CsCER6*, *CsCER7*

## Abstract

Fruit glossiness is an important external fruit quality trait that greatly affects the marketability of fresh cucumber (*Cucumis sativus*) fruits. A few reports have suggested that the extent of cuticular wax loading influences the glossiness of the fruit surface. In the present study, we tested the wax contents of two inbred cucumber lines, comparing a line with waxy fruit (3401) and a line with glossy fruit (3413). Wax content analysis and dewaxing analysis demonstrate that fruit cuticular wax loads negatively correlate with fruit glossiness in cucumber. Identifying genes that were differentially expressed in fruit pericarps between 3401 and 3413 and genes induced by abscisic acid suggested that the wax biosynthesis gene *CsCER6* (*Cucumis sativus ECERIFERUM 6*) and the regulatory gene *CsCER7* may affect wax accumulation on cucumber fruit. Expression analysis via RT-qPCR, GUS-staining, and in situ hybridization revealed that *CsCER6* and *CsCER7* are abundantly expressed in the epidermis cells in cucumber fruits. Furthermore, the overexpression and RNAi lines of *CsCER6* and *CsCER7* showed dramatic effects on fruit cuticular wax contents, fruit glossiness, and cuticle permeability. Our results suggest that *CsCER6* and *CsCER7* positively regulate fruit cuticular wax accumulation and negatively influence fruit glossiness.

## 1. Introduction

Cucumber (*Cucumis sativus* L.) is an important economic vegetable crop cultivated worldwide, whose fruits are consumed in processed form (primarily as pickles) or as fresh vegetables. For fresh cucumbers, external quality factors, such as fruit size, spines (trichomes), and warts, affect consumer choice and preference [1]. Among these traits, fruit glossiness has long been recognized as an important factor, as fruits with a glossy appearance are more popular than dull fruits [2,3,4]. Mizrach et al. developed a method for gloss evaluation of curved-surface fruits and vegetables, such as apple, eggplant, plum, and so on [3]. Fruit glossiness reflected by light reflectance spectra could be used for defining and parametrizing fruit freshness [5]. In Arabidopsis (*Arabidopsis thaliana*) and rice (*Oryza sativa*), mutants with defects in wax biosynthesis or deposition usually exhibit a bright appearance, indicating that the brightness of the plant surface is negatively correlated with the accumulation of wax on the epidermis [6,7,8]. In addition, it has been shown that the wax crystal structure may play a role in determining fruit glossiness [9]. In European plum, polishing on the fruit surface can improve fruit brightness by destroying the natural structure of cuticular wax [10].

The wax layers on plant surfaces are composed of two parts: an amorphous intra-cuticular wax layer embedded in cutin polymers and a lamellar epicuticular wax structure that coats the outer plant surface and gives it a whitish or dull appearance [11,12,13]. In most crops, epi-cuticular waxes are complex mixtures of very long-chain fatty acids (VLCFAs) and their derivatives, including acids, aldehydes, ketones, esters, primary and secondary alcohols, as well as alkenes [14]. Besides VLCFA derivatives, some species also contain cyclic compounds, which include aromatics and alicyclics [15,16].

The main location of wax biosynthesis is the endoplasmic reticulum (ER), and the pathway of wax biosynthesis in *Arabidopsis* stems is well characterized. Wax biosynthesis in *Arabidopsis* stems occur via two pathways: the decarbonylation pathway, which produces aldehydes, alkanes, secondary alcohols, and ketones, and the acyl reduction pathway, which yields primary alcohols and esters [14]. Multiple genes involved in wax biosynthesis have been identified through screens for mutants with wax defects. For example, *Arabidopsis eceriferum 1* (*cer1*) and *cer3* mutants have lower amounts of VLC alkanes and their derivatives relative to the wild type [17,18]. During precursor biosynthesis, CER6, a β-keto acyl-CoA synthase (KCS), and CER10, an enoyl-CoA reductase (ECR), participate in the biosynthesis of VLCFA from C24–C26 [19]. In the acyl reduction pathway, CER4 is an alcohol-forming fatty acyl-CoA reductase (FAR) that has specificity for VLCFAs and is responsible for the biosynthesis of primary alcohols in the epidermal cells of aerial tissues [20]. In addition, WSD1 (Wax Synthase/Diacylglycerol Acyltransferase 1) is required for stem wax ester biosynthesis in *Arabidopsis* [21], while MAH1 (Mid-chain Alkane Hydroxylase 1), a cytochrome P450 enzyme, functions in the formation of secondary alcohols and ketones in the decarbonylation pathway [22].

Factors that move waxes to the cell surface or that regulate the genes encoding enzymes and transport factors also affect wax accumulation. For example, the transport of waxy compounds through the ER–Golgi–plasma membrane–cell wall pathway is indispensable for the accumulation of waxes on the plant surface [14]. CER5, an ABC transporter localized at the plasma membrane of epidermal cells, exports waxes through the plasma membrane to the cell wall [23,24]. Moreover, several key factors regulate wax biosynthesis, for instance, CER7, WIN1 (WAX INDUCER1), and MYB96 [25,26,27]. The ribonuclease CER7 can regulate the transcript levels of *CER3* and *WAX2* to influence wax accumulation in *Arabidopsis* [25].

In cucumber, the molecular characterization of the relationship between wax and fruit glossiness remains obscure; only three genes have been suggested to contribute to fruit glossiness by regulating the accumulation of wax in the peel [28,29,30]. *CsCER1* and *CsWAX2* play important roles in VLC alkane biosynthesis in cucumber [28,29]. *CsDULL*, encoding a C2H2-type zinc finger transcription factor, may regulate the transcription of wax biosynthesis/transport genes in cucumber [30]. In this study, we determined that the accumulation of fruit cuticular waxes negatively influences the fruit pericarp brightness in cucumber by characterizing two inbred lines, 3401 with a waxy fruit surface and 3413 with a glossy fruit surface. Among the differentially expressed genes related to wax biosynthesis or deposition in fruit peels between the two inbred lines and genes induced by treatment with the phytohormone abscisic acid (ABA), we propose that CsCER6 and *CsCER7* participate in fruit cuticular wax accumulation in cucumber. Furthermore, we generated transgenic lines overexpressing *CsCER6* and *CsCER7* or with RNA interference (RNAi)-mediated knockdown of *CsCER6* and *CsCER7* to explore their functions in cuticular wax in cucumber. Our results show that *CsCER6* and *CsCER7* positively regulate fruit cuticular wax accumulation and negatively affect fruit glossiness.

## 2. Results

### 2.1. Fruit Cuticular Waxes Influence Fruit Brightness in Cucumber

**Figure 1:**
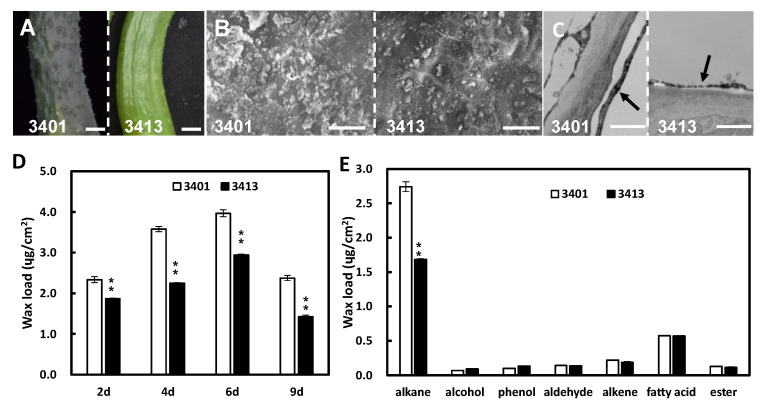
The phenotype of fruit cuticle in cucumber 3401 and 3413. (**A**) The fruit image of 3401 and 3413 at 9 days after anthesis (DAA). (**B**) The SEM pictures of the fruit cuticle, respectively, at 9 DAA in 3401 and 3413. (**C**) The TEM pictures of the fruit cuticle at 9 DAA in 3401 and 3413. The black arrow indicates wax layer. (**D**) Cuticular wax loads for fruit surface at 2, 4, 6, and 9 DAA in 3401 and 3413. (**E**) Contents of wax components in 3401 and 3413 at 6 DAA. The data of columns are the mean ± standard deviation. ** means *p* < 0.01, Student’s *t*-test. Scale bars = 1 cm in (**A**); 200 µm in (**B**); 1 µm in (**C**).

We selected two inbred lines 3401 and 3413, with significant differences in fruit glossiness for research in this study (Figure 1A). Compared to the waxy fruit surface of the 3401 line, fruit from the 3413 line exhibited a shiny appearance (Figure 1A). To investigate the cause of fruit glossiness, we inspected the fruit epidermis by scanning electron microscopy (SEM) and transmission electron microscopy (TEM). We observed more fruit cuticular wax crystalloids in 3401 than that in 3413 (Figure 1B); moreover, the fruit wax layer in 3401 was thicker than that in 3413 (Figure 1C). Wax content determination showed that the total wax loads per unit area of 3401 fruits were significantly higher than for 3413 at different developmental stages and reached a peak at 6 days after anthesis (DAA) (Figure 1D). We further analyzed wax composition at 6 DAA and determined that the cuticular waxes of both 3401 and 3413 fruits were composed of alkanes, fatty acids, alkenes, aldehydes, esters, phenols, and alcohols. Among these, alkanes were the main components of cuticular wax and differentially accumulated between 3401 (2.74 µg/cm^2^, 69% of total wax load) and 3413 (1.68 µg/cm^2^, 57% of total wax load) (Figure 1E). In previous studies, wax crystal structure could affect surface glossiness [9,10]. Therefore, we observed the structure of wax crystal on fruit surface of 3401 and 3413, and found that the wax crystal displayed irregular graininess both in 3401 and 3413, and the density of wax crystal gains increased in 3401, compared to that of 3413 (Figure 1B). It was consistent with the wax content between 3401 and 3413 (Figure 1D).

To validate the relationship between the contents of fruit cuticular waxes and cucumber fruit brightness, we dewaxed fruit at 9 DAA in 3401 by chloroform treatment. The dewaxed fruit (right) had an increased brightness relative to the control treated with water (left) (Figure S1A). Moreover, water droplets on dewaxed fruit (right) formed a film, in contrast to the typical water beads formed on regular fruit (left) via surface tension due to the cuticular wax layer (Figure S1B). These observations indicate that fruit cuticular waxes negatively influence fruit glossiness in cucumber.

### 2.2. Wax-Related Genes Are Differently Expressed between 3401 and 3413

**Figure 2:**
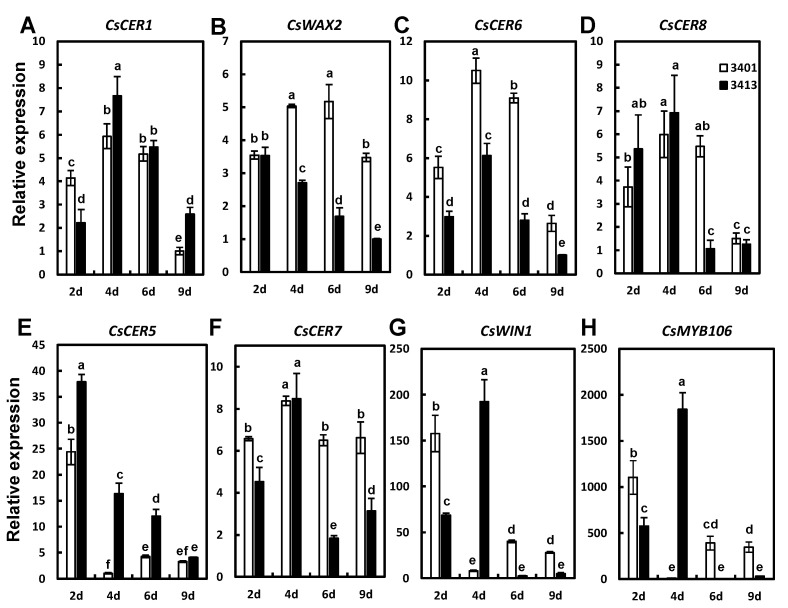
Relative expression of genes related to wax biosynthesis in different fruit developmental stages of 3401 and 3413. (**A**–**G**) Expression of *CsCER1* (**A**), *CsWAX2* (**B**), *CsCER6* (**C**), *CsCER8* (**D**), *CsCER5* (**E**), *CsCER7* (**F**), *CsWIN1* (**G**), and *CsMYB106* (**H**) at 2, 4, 6, and 9 DAA in 3401 and 3413. The data of columns are the mean ± standard deviation. The different lowercase letters indicate significant differences (*p* < 0.05) by one-way ANOVA analysis with the Dunnett test.

We examined the expression of wax-related genes in the pericarp of 3401 and 3413 fruit at different developmental stages (2, 4, 6, and 9 DAA), including the wax biosynthesis genes *CsCER1*, *CsWAX2*, *CsCER6*, and *CsCER8*, the wax transporter gene *CsCER5* and the wax biosynthesis regulator genes *CsCER7*, *CsWIN1*, and *CsMYB106*, by reverse transcription quantitative PCR (RT-qPCR) (Figure 2). Relative *CsCER1* expression levels in 3401 were higher than in 3413 only at 2 DAA (Figure 2A). The expression levels of *CsWAX2* in 3401 were significantly higher than those in 3413 at 4, 6, and 9 DAA (Figure 2B). *CsCER1* expression generally increased up to 4 DAA before declining, while *CsWAX2* expression in 3413 decreased across fruit development and was lower than in 3401. Similar to *CsCER1* and *CsWAX2* in 3401, the expression levels of *CsCER6*, *CsCER7*, and *CsCER8* also increased firstly before dropping, and *CsCER6* and *CsCER7* reached higher expression levels in 3401 than in 3413 at different developmental stages, consistent with the observed change for wax loads seen between 3401 and 3413 (Figure 2B–D,F). In both 3401 and 3413, *CsCER5* reached its highest expression level at 2 DAA and was expressed at a lower level in 3401 than in 3413, which was in contrast to the different wax loads of 3401 and 3413 (Figure 2E). The expression patterns of *CsWIN1* and *CsMYB106* did not exhibit a clear pattern during developmental stages in 3401 or 3413 (Figure 2G). Combined with the wax loads at different developmental stages, the expression results above indicate that *CsWAX2*, *CsCER6*, and *CsCER7* may be involved in the difference in fruit cuticular wax contents between 3401 and 3413.

### 2.3. ABA Can Induce Fruit Wax Deposition and the Expression of Wax Biosynthesis-Related Genes

ABA is an important phytohormone in plant responses to abiotic stress such as drought, high salt, and low temperature [31]. We observed that fruit total cuticular wax loads increased by 1.8-fold when 3413 fruits were treated with AB compared to water-treated fruits (Figure S2A). The expression of *CsCER1*, *CsCER6*, *CsWAX2*, *CsWIN1*, *CsCER8*, and *CsCER7* was significantly induced by ABA, with *CsCER7* being the most highly induced, as shown by RT-qPCR analysis (Figure S2B–I). Combined with the expression trends of the above genes in 3401 and 3413 (Figure 2), we speculated that *CsWAX2*, *CsCER6*, and *CsCER7* may play important roles in fruit cuticular wax deposition of cucumber. The function of *CsWAX2* has been studied in a previous study [29], thus *CsCER6* and *CsCER7* were chosen for further analysis in this study.

### 2.4. CsCER6 Positively Regulates Fruit Cuticular Wax Biosynthesis in Cucumber

**Figure 3:**
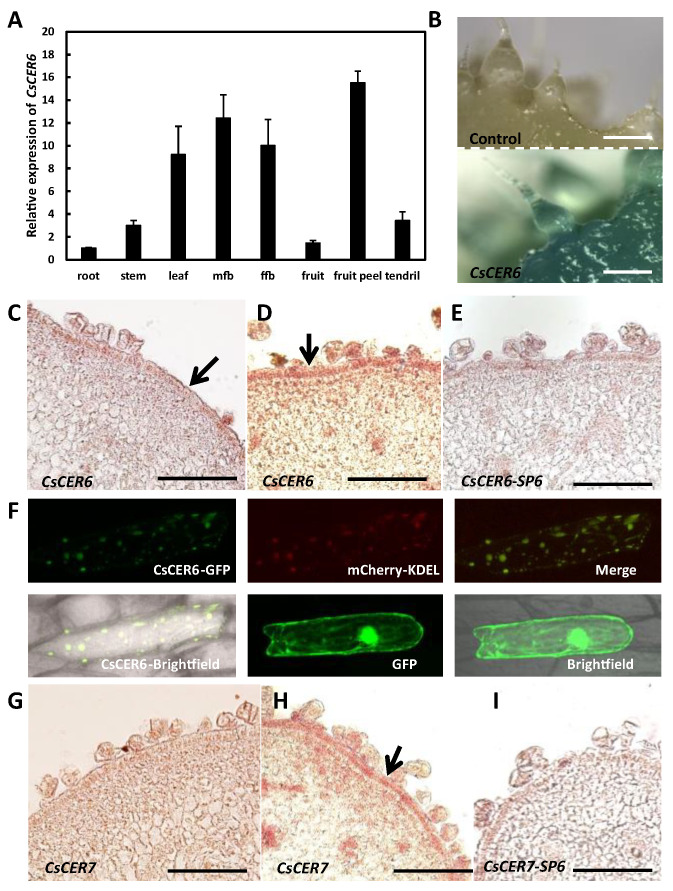
The expression pattern of *CsCER6* and *CsCER7*. (**A**) qRT-PCR analysis of *CsCER6* in various tissues in cucumber. The data of columns are the mean ± standard deviation. (**B**) Spatial expression pattern of *CsCER6* detected by a GUS reporter gene assay in cucumber fruit. (**C**–**E**) In situ hybridization of *CsCER6* (**C**,**D**) and *CsCER6*-sense probe (**E**) in 3461 fruits. (**C**) The fruit before anthesis; (**D**) the fruit at anthesis. (**F**) Subcellular localization of *CsCER6* protein in onion epidermal cells. (**G**–**I**) In situ hybridization of *CsCER7* (**G**,**H**) and *CsCER7*-sense probe (**I**) in 3461 fruits. (**G**), the fruit before anthesis; (**H**), the fruit at anthesis. mfb, male flower buds; ffb, female flower buds; mCherry-KDEL, ER (endoplasmic reticulum) marker with mCherry fluorescent protein. The arrows indicate epidermal cells in **C**–**D** and **H**. Scale bars = 1 mm in (**B**); 100 µm in (**C**–**E** and **G**–**I**).

To examine whether *CsCER6* might be involved in cuticular wax biosynthesis in cucumber fruits, we first characterized its expression pattern in various tissues. RT-qPCR analysis showed that *CsCER6* is expressed in root, stem, leaf, male flower bud, female flower bud, fruit, fruit peel, and tendril, with transcripts reaching its highest level in fruit peel (Figure 3A). We also generated cucumber lines harboring a transgene consisting of the ß-glucuronidase (GUS) reporter gene driven by the *CsCER6* promoter. We detected GUS staining in the fruit peel and spines of the *proCsCER6:GUS* cucumber transgenic lines (Figure 3B). We explored the *CsCER6* expression pattern in more detail by mRNA in situ hybridization. As shown in Figure 3C–E, *CsCER6* transcript signals accumulated in the fruit epidermis, and the signals before anthesis (Figure 3C) were weaker than at anthesis (Figure 3D).

The ER is the main site for wax biosynthesis [11]. To further examine the potential involvement of CsCER6 in wax biosynthesis, we investigated its subcellular localization with a construct encoding a CsCER6-GFP (green fluorescent protein) fusion protein. We detected green fluorescence that co-localizes with the ER marker mCherry-KDEL, whereas the 35S:GFP control showed green fluorescence throughout the onion epidermal cell (Figure 3F). Hence, these data further suggested that CsCER6 is likely to be involved in cuticular wax biosynthesis in cucumber fruit.

**Figure 4:**
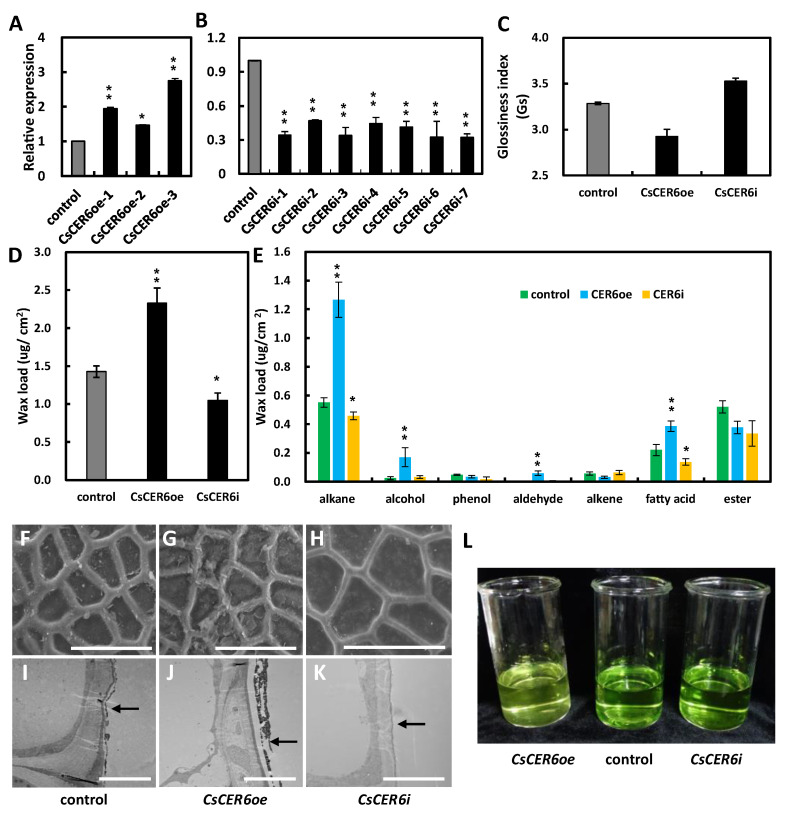
*CsCER6* regulates cucumber wax content in cucumber. (**A**,**B**) qRT-PCR analyses of *CsCER6* in control, various *CsCER6* overexpressing (**A**) and *CsCER6*-RNAi lines (**B**). (**C**) Fruit glossiness value of 9 DAA fruit in control, *CsCER6i*, and *CsCER6oe* lines. (**D**) The cuticular wax load of 9 DAA fruit in control, *CsCER6oe*, and *CsCER6i* lines. (**E**) The cuticular wax composition of 9 DAA fruit in control, *CsCER6oe* and *CsCER6i* lines. The data of columns are the mean ± standard deviation. (**F**–**H**) Epicuticular wax crystal formation on 9 DAA fruit surfaces of control (**F**), *CsCER6oe* (**G**) and *CsCER6i* plants by SEM. (**I**–**K**) The wax layer on 9 DAA fruit surfaces of control (**I**), *CsCER6oe* (**J**), and *CsCER6i* (**K**) plants by TEM. The arrows indicate the wax layer. (**L**) Chlorophyll permeation test of different transgenic CsCER6 cucumber lines. * means *p* < 0.05, ** means *p* < 0.01, Student’s *t*-test. Scale bars = 40 µm in (**F**–**H**); 1 µm in (**I**–**K**).

To verify the role of *CsCER6* in wax biosynthesis, we constructed three overexpression and seven RNAi transgenic lines. *CsCER6* expression was 1.3- to 2.7-folds higher in the overexpression lines (*CsCER6oe*) and decreased by at least 50% in RNAi lines (*CsCER6i*) compared to the control plants (Figure 4A,B). Then *CsCER6oe-3,* with the highest expression, and *CsCER6i-7,* with the lowest expression, were chosen for further phenotype analysis, with wild-type plants as control.

A fruit glossiness value test showed that *CsCER6i* had a glossy fruit phenotype, in contrast to the waxy fruit of *CsCER6oe* lines compared to the control (Figure 4C). We further measured the fruit cuticular wax contents of the transgenic lines, revealing that the wax load per unit area of *CsCER6oe* and *CsCER6i* was 2.33 and 1.05 µg/cm^2^, respectively, which corresponded to 163.1% and 73.4% of that in control plants, respectively (Figure 4D). Among several wax components, alkene (1.27 µg/cm^2^), alcohol (0.17 µg/cm^2^), aldehyde (0.59 µg/cm^2^), and fatty acids (0.39 µg/cm^2^) were markedly higher than control in *CsCER6oe*, and the proportion of alkane contents in total wax loads increased from 38.7% in the control lines to 54.4% in *CsCER6oe* (Figure 4E). In *CsCER6i*, alkenes and fatty acids significantly decreased by 16.9% and 38.2%, respectively, relative to the control (Figure 4E). SEM pictures of the fruit surface in *CsCER6oe* indicated larger wax crystals on the fruit surface, while TEM images showed a thicker wax layer in *CsCER6oe* compared to that of control lines, whereas the wax layer of *CsCER6i* was thinner (Figure 4F–K).

Cuticular wax can influence plant cuticle permeability [32], which prompted us to measure chlorophyll leaching in the control and transgenic lines. Compared to the control line, chlorophyll a and b were extracted more slowly from *CsCER6oe* and faster from *CsCER6i* fruits (Figure 4L and Figure S3A,B). The above data suggest that *CsCER6* plays an important role in the cuticular wax biosynthesis of cucumber fruit.

### 2.5. CsCER7 Positively Regulates Fruit Cuticular Wax Biosynthesis in Cucumber

**Figure 5:**
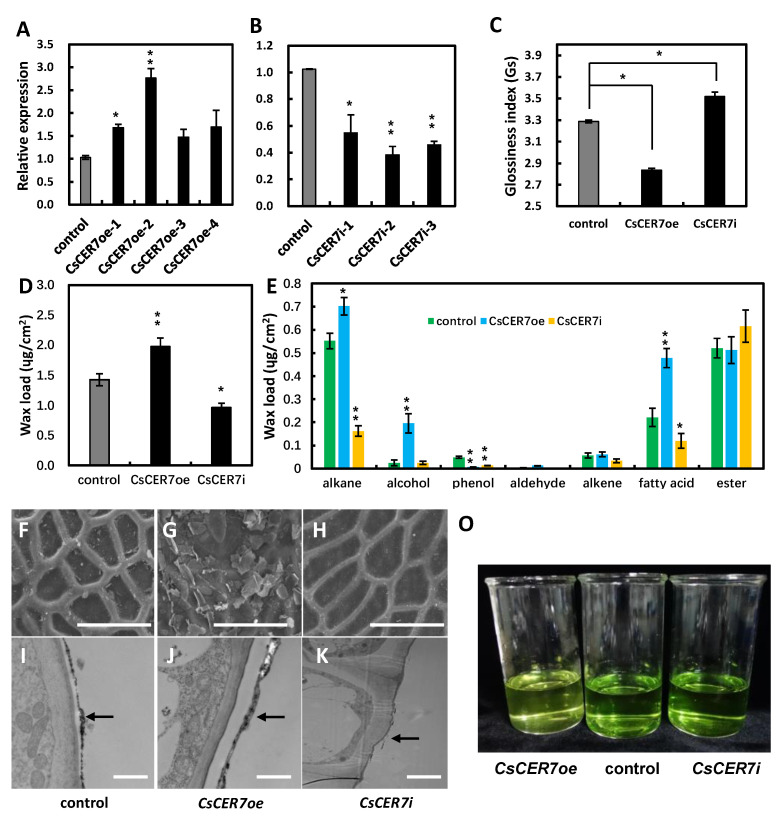
*CsCER7* regulates cucumber wax content in cucumber. (**A**,**B**) qRT-PCR analyses of *CsCER7* in control, various *CsCER7* overexpressing (**A**) and *CsCER7*-RNAi lines (**B**). (**C**) Fruit glossiness value of 9 DAA fruit in control, *CsCER7i*, and *CsCER7oe* lines. (**D**) The cuticular wax load of 9 DAA fruit in control, *CsCER7oe*, and *CsCER7i* lines. (**E**) The cuticular wax composition of 9 DAA fruit in control, *CsCER7oe*, and *CsCER7i* lines. The data of columns are the mean ± standard deviation. (**F**–**H**) Epicuticular wax crystal formation on 9 DAA fruit surfaces of control (**F**), *CsCER7oe* (**G**), and *CsCER7i* (**H**) plants by SEM. (**I**–**K**) The wax layer on 9 DAA fruit surfaces of control (**I**), *CsCER7oe* (**J**), and *CsCER7i* (**K**) plants by TEM. The arrows indicate the wax layer. (**O**) Chlorophyll permeation test of different transgenic *CsCER7* cucumber lines. * means *p* < 0.05, ** means *p* < 0.01, Student’s *t*-test. Scale bars = 40 µm in (**F**–**H**); 1 µm in (**I**–**K**).

mRNA in situ hybridization results showed that *CsCER7* is expressed specifically in fruit epidermal cells, with stronger signals in fruit at anthesis than before anthesis (Figure 3G–I), suggesting that *CsCER7* is likely to play a role in fruit epidermal cells of cucumber. As with *CsCER6* above, we obtained four overexpression (*CsCER7oe*) and three RNAi (*CsCER7i*) transgenic lines. Relative *CsCER7* transcript levels were 1.7- and 2.6-fold higher than control in *CsCER7oe-1* and *CsCER7oe-2*, respectively (Figure 5A), and were at least 40% lower in RNAi lines: 41% in *CsCER7i-1*, 61% in *CsCER7i-2*, 55% in *CsCER7i-3* (Figure 5B). we thus selected *CsCER7oe-2* and *CsCER7i-2* for phenotypic analysis, with wild-type plants serving as control.

Compared to the control, the fruit glossiness value of *CsCER7oe* decreased and that of *CsCER7i* increased (Figure 5C). The analysis of wax composition showed that *CsCER7oe* and *CsCER7i* have wax loads of 1.97 and 0.97 µg/cm^2^, respectively, equivalent to 138.0% and 68.1% of control, respectively (Figure 5D). Compared to the control, the contents of alkanes (0.70 µg/cm^2^), alcohols (0.20 µg/cm^2^), and fatty acids (0.48 µg/cm^2^) in *CsCER7oe* were markedly higher than the control, while the contents of alkanes (0.16 µg/cm^2^) and fatty acids (0.12 µg/cm^2^) significantly lower than the control in *CsCER7i* (Figure 5E). In SEM pictures, compared to control plants, we observed more and less wax crystals on the fruit surface in *CsCER7oe* and *CsCER7i* lines, respectively, compared with the control (Figure 5F–H). Moreover, the fruit cuticular wax layer was thicker and thinner in *CsCER7oe* and *CsCER7i* lines, respectively, by TEM (Figure 5I–K). In addition, *CsCER7oe* and *CsCER7i* exhibited slower and faster chlorophyll a and b leaching rates, respectively (Figure 5O and Figure S3C,D). These above data indicate that *CsCER7* is also involved in regulating cuticular wax loads of cucumber fruit.

## 3. Discussion

### 3.1. Fruit Cuticular Wax Loads Are Negatively Correlated with Fruit Glossiness in Cucumber

A layer of cuticular wax, forming a first physical barrier, is deposited on the surface of terrestrial plants, which reduces the various types of damage caused by environmental stress and maintains growth in a relatively stable internal environment [33,34,35]. In addition to this structural role, cuticular wax has been proposed to influence the glossiness of the plant surface [6,7,36]. The wax defect mutants usually exhibit a more glossy appearance than their wild-type plants [18,34,36,37,38]. For instance, the wax load per unit of leaf area of the *Arabidopsis cer1-1* and *cer1-2* mutants is significantly lower than in wild-type plants, resulting in a bright green appearance [18]. Likewise, the significant decrease in wax content seen in the *glossy1* mutant in rice is accompanied by glossy leaves [33]. Mature fruits from mutants with lower wax loads are much glossier than the wild-type “Newhall” in navel orange (*Citrus sinensis* [L.] Osbeck cv. Newhall) [36]. Except for wax content, the wax crystal structure was involved in affecting the glossiness appearance [9,10,13]. In kohlrabi, the plant with a whitish surface had more wax loads and a different wax crystal structure compared to that with a glossy surface [13]. In European plum, polishing the fruit surface can improve the fruit’s brightness by destroying the cuticular wax’s natural structure [10].

In cucumber, fruit glossiness is an important external quality trait affecting the marketability of fresh-consumed cucumber fruits. Although several works have characterized cuticular wax in cucumber, the exact relationship between fruit cuticular wax and fruit glossiness has been ambiguous [28,29,30]. Wang et al. found that RNAi lines of *CsCER1* and *CsWAX2* influenced the wax properties and exhibited a glossy fruit phenotype in cucumber [28,29]. *CsDULL* is a candidate gene for *glossy fruit* lines with a defective cuticle [30]. In this study, we showed that the fruits in the inbred line 3401 with a dull fruit surface have more cuticular wax contents than inbred line 3413 with glossy fruit (Figure 1). The density of wax crystals on the fruit surface and the thickness of the cuticular wax layer were lower in 3413 compared to 3401, consistent with the wax content test result (Figure 1). In addition, the wax crystal structure displayed irregular graininess both in 3401 and 3413 (Figure 1B), suggesting that the structure of cuticular wax crystal may not be involved in the fruit glossiness between 3401 and 3413 in cucumber. The further dewaxing test showed that the surface of dewaxed fruit exhibited a brighter appearance (Figure 1F,G). These data suggested that fruit cuticular wax contents may negatively influence fruit glossiness in cucumber.

Besides, different vegetable or fruit species had different cuticular wax content. For example, the wax content of four cabbage lines with different gloss ranged from about 20–40 µg/cm^2^ [39]; in tomato, the *woolly* mutant and its wild-type lines had about 8 and 24 µg/cm^2^ wax load of fruits, respectively [40]; The total wax amounts of mature fruits were highly variable among 12 grape cultivars, ranging from 4.79 to 20.57 µg/cm^2^ [9]. In this study, we detected the fruit cuticular wax loads ranging from 1.42 to 3.97 ug/cm^2^ between 3401 and 3413 (Figure 1). It suggested that the wax content of different vegetable species and different cultivars of the same species varied greatly, and the fruit cuticular wax content in cucumber was relatively less among vegetable or fruit species.

### 3.2. ER-Localized CsCER6 Plays an Important Role in Wax Biosynthesis in Cucumber Fruits

VLCFAs are substrates for the production of cuticular waxes, and their biosynthesis is controlled by the activity of β-ketoacyl-CoA synthase enzymes (condensing enzymes) and fatty acid elongases, which determine the amounts of fatty acid products during the elongation process [41,42]. The condensing enzyme CER6 in *Arabidopsis* and its homologs in other species displayed a conserved function in wax biosynthesis [17,43]. In *Arabidopsis*, the specific expression pattern of *CER6* in the epidermis of aerial organs is thought to be an important factor in the control of wax accumulation [19]. In this study, we screened *CsCER6* from the differentially expressed genes between inbred lines 3401 with a waxy cuticle and 3413 with a waxless cuticle, as well as following an ABA treatment (Figure 2 and Figure S2). *CsCER6* was expressed to higher levels in the fruit peel than that in whole fruits by RT-qPCR, which was consistent with the GUS staining results obtained with *CsCER6pro:GUS* reporter line and mRNA in situ hybridization results (Figure 3A–E). In addition, CsCER6 co-localized with the ER marker mCherry-KEDL in the ER (Figure 3F). The ER is the main site of wax biosynthesis in plants [14,44].

Overexpression and RNAi lines of *CsCER6* produced more and less fruit cuticular waxes compared to the control lines, respectively, validating that CsCER6 plays an important and conserved role in cuticular wax biosynthesis in cucumber. The glossy fruits of *CsCER6* RNAi lines with lower wax loads, together with the dull fruits with more wax loads of *CsCER6* overexpression lines (Figure 4) indicated that *CsCER6* negatively affected fruit glossiness via positively influencing fruit cuticular wax biosynthesis in cucumber. In addition, chlorophyll leaching tests of fruits suggested that fruit cuticular wax may affect fruit shelf life by affecting fruit epidermal permeability.

### 3.3. CsCER7 Is Involved in Regulating Fruit Cuticular Wax Contents in Cucumber

In Arabidopsis, the *cer7* mutant exhibits reduced cuticular wax accumulation and considerably lower expression levels of *CER3*, a key wax biosynthetic gene [25,45]. *CER7* encodes a 3′-5′ exoribonuclease and is the core subunit of the exosome, which is thought to modulate the levels of the transcriptional repressor *CER3* mainly by degrading the *CER3* mRNA [46,47]. In maize (*Zea mays*), a CER7 homolog was identified by a genome-wide association study for the cuticular conductance of adult leaves, and is potentially involved in cuticle biosynthesis [48]. In this study, we discovered that CsCER7, the CER7 homolog in cucumber, was abundantly expressed in the fruit epidermis, indicating *CsCER7* may be involved in epidermis function (Figure 3G–I). The *CsCER7* RNAi transgenic lines exhibited glossy fruits with lower wax loads, while *CsCER7* overexpression lines showed a waxy fruit surface with more wax loads compared to the control (Figure 5), suggesting that *CsCER7* positively regulates fruit cuticular wax content in cucumber.

## 4. Materials and Methods

### 4.1. Plant Material and Growth Conditions

Cucumber inbred lines 3401, 3413, and 3461 were used in this study; 3401 and 3413 were used for expression analysis of various wax biosynthesis-related genes, while 3461 was used for constructing transformation lines. All cucumber lines were incubated at 25/18 °C (day/night) under 16/8 h (light/dark) until the three true-leaf stage, then planted in a greenhouse located at Beijing Jinliuhuan agricultural farm. Before being planted in the greenhouse, organic compost and three-nutrient compound fertilizers (among the nitrogen fertilizer is ammonium nitrogen) were uniformly applied to the soil. Pest and water control were carried out according to standard protocols. For ABA treatment, fruits in 3413 plants at the fruit-bearing stage were sprayed with ABA solution (200 µM) twice on the day before flowering and on the day of flowering, respectively. The control fruits were sprayed with water at the same time. Then three fruits from the treatment and control group at 6 days after anthesis (DAA) were used for wax analysis and gene expression analysis.

### 4.2. Scanning Electron Microscopy (SEM) and Transmission Electron Microscopy (TEM) Analysis

Pericarps of the middle part of fruits at 6 and 9 DAA were used for SEM and TEM analysis. For SEM analysis, samples were fixed with 2.5% (*w*/*v*) glutaraldehyde, washed with PBS (pH 7.2), and post-fixed in 1% (*v*/*v*) OsO4. Then, the samples were dehydrated through ethanol series, critical-point dried, and coated with gold–palladium (EIKO IB-3, Ion Coater, Tokyo, Japan) [49]. Images were taken with a Hitachi S-4700 scanning electron microscope using a 2 kV accelerated voltage. For TEM analysis, the samples fixed by 2.5% (*w*/*v*) glutaraldehyde were prepared as described [50]. Thin sections were cut with a LEICA UC6I microtome (Leica, Weztlar, German), then imaged with a JEM-123O scanning transmission electron microscope (JEOL, Tokyo, Japan).

### 4.3. Wax Content Test

The pericarp of the middle part of the fruit was used for the wax content test. Cucumber cuticular wax was extracted from fruit peel by immersing the 2 cm^2^ epicarp in chloroform at room temperature for 1 min, then immersed in chloroform at 60 °C for 2 min. After mixing the solutions, an internal standard docosane was added and the samples were dried under N_2_ gas. Then the fruit cuticular wax was analyzed by gas chromatography–mass spectrometer (GC–MS) described by Bourdenx et al. [18]. Three biological samples were performed.

### 4.4. Transcript Abundance Analysis

Real-time quantitative PCR (RT-qPCR) was employed for measuring transcript abundances. Total RNA was extracted from the young root, stem, leaf, male flower buds, female flower buds, fruit at anthesis, fruit pericarps, and tendril (Tiandz, http://www.tiandz.com, accessed on 6 May 2016), then reverse-transcribed by the PowerScriptTM reverse transcriptase (Invitrogen, Carlsbad, America). The ID number of the genes for RT-qPCR are listed in Supplemental Table S1. The PCR amplification was performed on Applied Biosystems 7500 real-time PCR systems using SYBR Premix Ex Taq (TaKaRa, Osaka, Japan), with gene-specific primers listed in Supplemental Table S2. The cucumber α-TUBULIN (TUA) gene was used as a reference control to normalize the expression data. RT-qPCR were repeated in three biological samples and the 2^−∆∆Ct^ method was used for data analysis. The primers used are listed in Supplemental Table S2.

### 4.5. Subcellular Localization

The full-length coding region without stop codon of CsCER6 was inserted between the *KpnI* and *BamHI* sites of the *pEZS-NL* vector to generate *35S:CsCER6-GFP*; the empty *pEZS-NL* vector was used as the negative control. Then, the *35S:CsCER6-GFP* construct and ER marker mCherry-KDEL [32] were co-introduced into onion epidermal cells using a pneumatic particle gun (Model PDS-1000/He; BIORAD, Beijing, China) as described previously [51]. The fluorescence signals were detected using the confocal laser-scanning microscope (Nikon C1, Tokyo, Japan).

### 4.6. Cucumber Transformation

For the β-glucuronidase (GUS) staining analysis, a 2000 bp fragment upstream of the start codon of *CsCER6* was inserted into *pCAMBIA1391* vector between *SpeI* and *NcoI* to generate *proCsCER6:GUS* vector. For function research of *CsCER6* and *CsCER7*, the overexpression and RNAi vectors were constructed with *PBI121* and *pFGC1008*, respectively. The full-length coding region of *CsCER6* and *CsCER7* were inserted into *PBI121* between *XbaI* and *SmaI* under 35S promoter, respectively. The RNAi vector (*CsCER6-pFGC1008* and *CsCER7-pFGC1008*) were generated as described previously [52]. The primers used were listed in Supplemental Table S2.

The *proCsCER6:GUS*, *CsCER6-PBI121*, *CsCER6-pFGC1008*, *CsCER7-PBI121*, and *CsCER7-pFGC1008* were transformed into the Agrobacterium tumefaciens strain CS58 by electroporation method, then transformed into cucumber cultivar 3461 using the cotyledon transformation method, as described previously [52].

### 4.7. GUS Staining

(GUS) staining assays were performed according to the protocol described [51]. The young fruits at anthesis were fixed and incubated in GUS-staining solution for 24 h at 37 °C, then cleaned with 80% ethanol and imaged.

### 4.8. In situ Hybridization

Fruits at anthesis were fixed by 3.7% FAA. Specific sequence fragments of *CsCER6* and *CsCER7* were amplified with specific primers using SP6 and T7 polymerase for sense and antisense probes, respectively. Sample fixation, sectioning, and in situ hybridization were performed as described previously [53].

### 4.9. Chlorophyll Leaching Assay

Pericarps in the middle of fruits (2 cm in length and 3 cm in diameter) at 9 DAA were collected and immersed in equal volumes of 200 mL 80% ethanol in the dark. Every 20 min, 1 mL extract solution was used for absorbance test at 645 nm and 633 nm, in all for six times. Lambert–Beer law was used for calculating the extraction concentration of chlorophyll a and b at different time points; the calculation formula was Ca = 12.7*A663-2.69*A745 and Cb = 22.9*A645-4.68*A633, and then drawn the chlorophyll extraction rate curve [54]. Three biological samples were performed.

## 5. Conclusions

In this study, we demonstrated that fruit cuticular wax loads per unit area are negatively correlated with fruit glossiness in cucumber by characterizing two inbred lines with opposite wax contents and by performing a dewaxing test. We showed that *CsCER6* and *CsCER7* negatively influence fruit glossiness by positively regulating fruit cuticular wax accumulation. Overall, our findings will provide theoretical support and gene resources for the comprehensive improvement of cucumber fruit appearance quality in future cucumber breeding efforts.

## Data Availability

Data used in this study are presented in the article or supplementary information.

## Supplementary Materials

The following supporting information can be downloaded at: https://www.mdpi.com/article/10.3390/ijms24021135/s1.
