# Supplementary material for: CsCER6 and CsCER7 Influence Fruit Glossiness by Regulating Fruit Cuticular Wax Accumulation in Cucumber
Source: Int J Mol Sci. 2023 Jan 6;24(2):1135. doi: 10.3390/ijms24021135 (PMC9864978; doi:10.3390/ijms24021135)
Supplement: Supplementary file 1 [file ijms-24-01135-s001.zip › ijms-2082337-supplementary.pdf]

Supplementary Figure S1

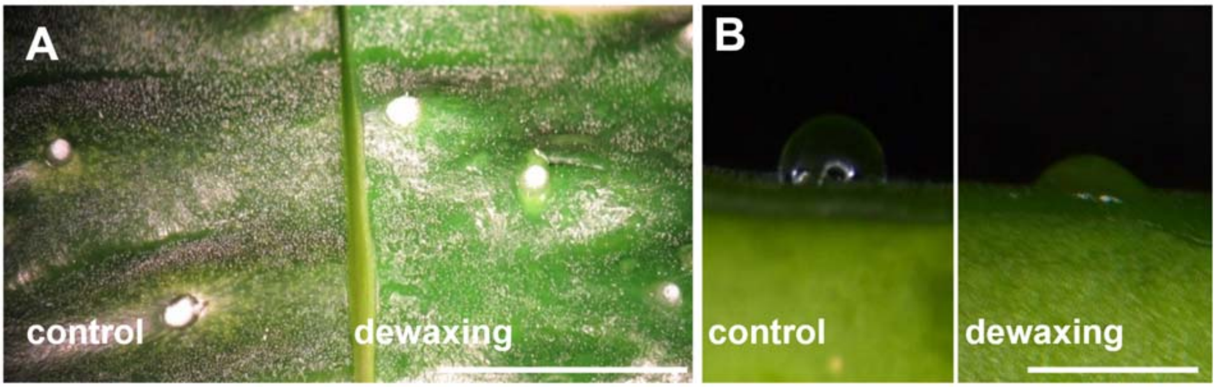

Figure S1. The phenotype of fruit cuticle in cucumber 3401 after dewaxing. (A)The brightness of fruit peel after immersed in water (left) or chloroform (right). (B) The behaviour of water droplets on the 9 DAA 3401 fruit peel after immersed in water (left) or chloroform (right). Scale bars=1cm in A and B.

Supplementary Figure S2

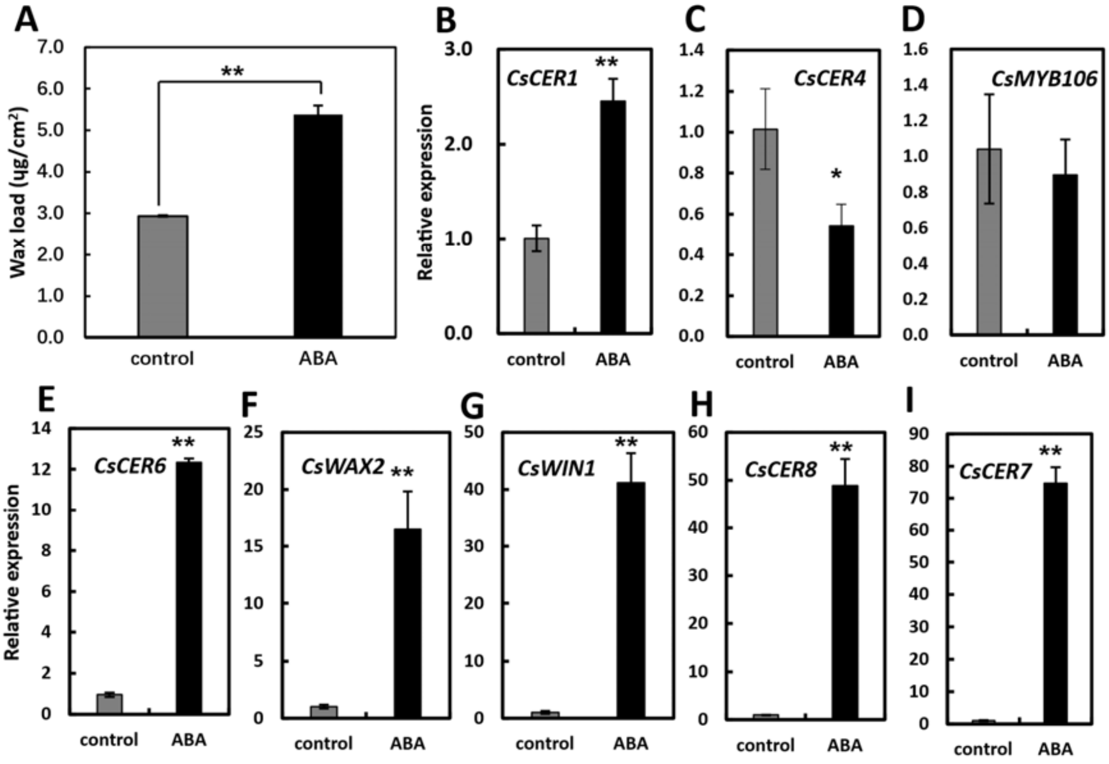

Figure S2. ABA-treatment causes the changes of wax loads (A) and the transcription level of wax related genes (B-I). The data of columns are the mean ± standard deviation. * means $p < 0.05$, ** means $p < 0.01$, Student's t-test.

Supplementary Figure S3

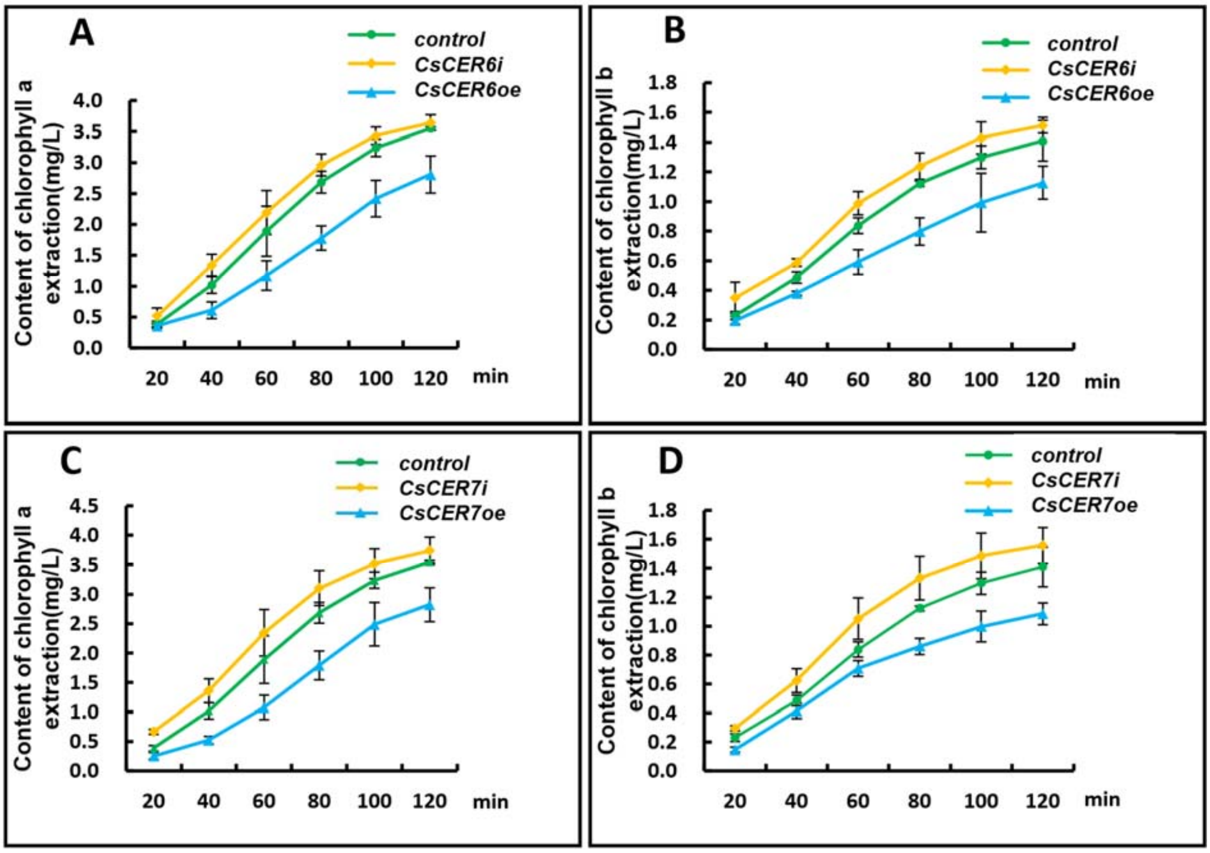

Figure S3. Chlorophyll leaching assay with cucumber fruits at 9 DAA from control plants, *CsCER6*-(A-B) and *CsCER7*-transgenic lines (C-D). The data of lines are the mean ± standard deviation.

Supplemental Table S1. The gene ID list in this study.

Gene Name	Gene ID
CsCER6	*Csa001017*
CsCER7	*Csa006488*
CsCER1	*Csa024936*
CsWAX2	*Csa020530*
CsCER5	*Csa007789*
CsCER8	*Cucsa.300670*
CsWIN1	*Csa006854*
CsCER4	*Csa015124*

Supplemental Table S2. Primers used in this study.

**Primers for gene cloning**	
CsCER6-clone-F	ATGCCTCCAATCTTGCCGG
CsCER6-clone-R	TTAGAGTTTGACTACTTCAGG
CsCER7-clone-F	ATGGAGCAGAGATTAGCCAA
CsCER7-clone-R	CTAATCCACATCGATAGGGGT
**Primers for qRT-PCR**	
CsCER1-qRT-F	CTACACTCCCTCGTTCCATTCA
CsCER1-qRT-R	TGCCAACCCAAGCCTCATA
CsWAX2-qRT-F	GCAGAATAGCTTAAATGCAGGC
CsWAX2-qRT-R	TACAATGAAGGCAGCGGGC
CsCER6-qRT-F	CTCATCCCTGTCATGCTCGC
CsCER6-qRT-R	GACAGTGGCAACGAAGATGATG
CsCER7-qRT-F	AAATGATGTCCTCTGAGTCTATGGTT
CsCER7-qRT-R	CGATAGGGGTGGTGGTCTTC
CsCER8-qRT-F	AAAAATCCTGGAAACCGTATGC
CsCER8-qRT-R	ATTCTATGGCACCAGCACCTAA
CsWIN1-qRT-F	TCATTTCCCCATCTTCTCCG
CsWIN1-qRT-R	CATCCATTTCCAACTTCCTCAT
CsMYB106-qRT-F	TTCACTCAAAAAACATCAACAAACT
CsMYB106-qRT-R	AATCCTTCTCCTATTCCTCCACTAC
TUA-qRT-F	ACGCTGTTGGTGGTGGTAC
TUA-qRT-R	GAGAGGGGTAAACAGT
**Primers for gene vector construction**	
CsCER6-gus-F	GCTCTAGAGAGAAAATTGAAGGTTAAGGGC
CsCER6-gus-R	TCCCCCGGGGAGAGAGATGTAAGAATGAATGGG
CsCER6-gfp-F	GCTCTAGAATGCCTCCAATCTTGCCG
CsCER6-gfp-R	TCCCCCGGGGAGTTTGACTACTTCAGGAATGTGAA
CsCER6-oe-F	GCTCTAGAATGCCTCCAATCTTGCCG
CsCER6-oe-R	TCCCCCGGGTTAGAGTTTGACTACTTCAGGAATGTGA
CsCER6i-for-F	TTGGCGCGCCTCTCGTCAACCACATTTTAACTCT
CsCER6i-for-R	ATTTAAATCATCTGAAACTCAACGCTCTTG
CsCER6i-rev-F	GGACTAGTTCTCGTCAACCACATTTTAACTCT
CsCER6i-rev-R	CGGGATCCCATCTGAAACTCAACGCTCTTG
CsCER7-oe-F	GCTCTAGAATGGAGCAGAGATTAGCCAATAC
CsCER7-oe-R	TCCCCCGGGCTAATCCACATCGATAGGGGTG
CsCER7i-for-F	TTGGCGCGCCGATATCACAACTAAAATAAAAAATGCAG

CsCER7i-for-R	ATTTAAATGCGGTTAGATTTCCACGTGA
CsCER7i-rev-F	GGACTAGTGATATCACAACTAAAATAAAAAATGCAG
CsCER7i-rev-R	CGGGATCCGCGGTTAGATTTCCACGTGA
**Primers for *in situ* hybridization**	
CsCER6-SP6	GATTTAGGTGACACTATAGAATGCTAACTTGGCTACCAGTATCTCGTC
CsCER6-T7	TGTAATACGACTCACTATAGGGCTCAAGAATCCTCATCTGAAACTC
CsCER7-SP6	GATTTAGGTGACACTATAGAATGCTAGTTAGGGCGTGTAATAGACCG
CsCER7-T7	TGTAATACGACTCACTATAGGGTGACAGAAGCAATTCGCAGAC
